# Fumigant Toxicity and Oviposition Deterrency of the Essential Oil from Cardamom, *Elettaria cardamomum*, Against Three Stored—product Insects

**DOI:** 10.1673/031.011.16501

**Published:** 2011-12-04

**Authors:** Habib Abbasipour, Mohammad Mahmoudvand, Fahimeh Rastegar, Mohammad Hossein Hosseinpour

**Affiliations:** ^1^ Department of Plant Protection, Faculty of Agricultural Sciences, Shahed University, Tehran, Iran; ^2^ Department of Plant Protection, Khorramabad Branch, Islamic Azad University, Khorramabad, Iran

**Keywords:** *Callosobruchus*, *Ephestia*, *Tribolium*

## Abstract

Use of insecticides can have disruptive effects on the environment. Replacing the chemical compounds in these insecticides with plant materials, however, can be a safe method with low environmental risk. In the current study, chemical composition and insecticidal activities of the essential oil from cardamom, *Elettaria cardamomum* L. (Maton) (Zingiberales: Zingiberaceae) on the adults of three stored product pests was investigated. Results indicated that essential oil of *E. cardamomum* toxic to the bruchid beetle, *Callosobruchus maculatus* Fabricius (Coleoptera: Bruchidae), the red flour beetle, *Tribolium castaneum* Herbst (Coleoptera: Tenebrionidae), and the flour moth, *Ephestia kuehniella* Zeller (Lepidoptera: Pyralidae). Adults of *E. kuehniella* were more sensitive than the Coleoptera. Also, the highest mortality of these insects was seen after 12 hours. Results of the LT50 tests showed that the lethal time of mortality occurred between 10–20 hours in various test concentrations. Essential oil of *E. cardamomum* had a good efficacy on oviposition deterrence of *C.* *maculatus* females, too. The chemical constituents of the essential oils were analyzed by gas chromatography—mass spectrometry. The major constituents of cardamom were identified as 1,8-cineol, α-terpinyl acetate, terpinene and fenchyl alcohol. These results suggest that essential oil of *E. cardamomum* is a good choice for control of stored product pests.

## Introduction

The use of synthetic insecticides in agricultural pest control practices to increase yields and protect stored products may involve serious health hazards for organisms in the class Mammalia. These insecticides are often associated with residuals that are dangerous for the consumer and the environment. In addition, the risk of developing insect resistance and the high cost—benefit ratio of synthetic pesticides pushes research towards investigating alternative insecticides.

Aromatic plants, which are widespread in the rich and diversified flora of Iran, are known for their nutritional and medicinal characteristics. They are often found in cosmetics, perfumes, detergents, as well as in industries such as pharmacology and food flavoring. In addition to antiseptic and fungicidal properties identified in many plants (Benjilali et al. 1984; Caccioni and Guizzardi 1994; Dayal and Purohit 1971; Ghfir et al. 1997; Mishra and Dubey 1994), it has been shown that many aromatic plant species and their essential oils present a high level of efficiency in protecting crops and stored food against insect pests (Arnason et al. 1989; Bernard et al. 1995; Don-Pedro 1996; Mahmoudvand et al. 2011a, 2011b; Regnault-Roger and Hamraoui 1993, 1995; Saxena 1989). Plant material can also be effective as oviposition deterrents (Abbasipour et al. 2010) and antifeedants (Abbasipour et al. 2011). Essential oils have potential to be developed for use in insect control programs.

The cardamom, *Elettaria cardamomum* L. (Maton) (Zingiberales: Zingiberaceae), called the “Queen of Spices” in India, is a tall, perennial, reed—like herb that grows wild and is cultivated in India and Sri Lanka. Cardamom is an important economic crop and is a highly valued spice (Parthasarathy et al. 2008). The essential oils have therapeutic benefits (Lewis 1984; Nirmala 2000). Some studies showed that cardamom essential oils have inhibitory effects against fungus growth (Utta-U'r et al. 2000) and marked antiapamodic, analgesic, and anti—inflammatory activity (Al-Zuhair et al. 1996).

The characteristic flavors and odors emanating from the volatile oils of spices are known to have various effects on insect pests, including stored insects (Jacobson 1989; Shayya et al. 1991). The volatile oil from cardamom acts as a potential grain protectant by killing various life stages of the stored product insects attacking wheat, such as *Tribolium castaneum* and *Sitophilus zeamais*, via contact and fumigant action (Huang et al. 2000). Kim et al. (2004) worked on the acaricidal activity of 56 plant essential oils on the poultry red mite, *Dermanyssus gallinae*, and found that cardamone ceylon oil caused 100% mortality on adult mites.

This study was undertaken to investigate *E. cardamomum* essential oil composition. In addition, this paper describes for the first time the fumigation activity of the oil against three important stored products pests: the bruchid beetle, *Callosobruchus maculatus* Fabricius (Coleoptera: Bruchidae), the red flour beetle, *Tribolium castaneum* Herbst (Coloeptera: Tenebrionidae) and the flour moth, *Ephestia kuehniella* Zeller (Lepidoptera: Pyralidae). The effect of essential oils on oviposition deterrence of *C. maculatus* was also evaluated.

## Materials and Methods

### Extraction of the essential oil

The seeds of *E. cardamomum* were collected October 2009 for essential oil extraction. Samples were shade dried at room temperature and were subjected to hydrodistillation using a Clevenger type apparatus (Cavalcanti et al. 2004). For extraction of essential oils, 50 g of air—dried seed material in water (1:12 w:v) was used, with a distillation time of 4 hours. The oils were dried over anhydrous sodium sulphate and stored at 4 °C in the dark. Essential oil yield was 4–7%.

### Gas chromatography—mass spectrometry analysis

The oil was analyzed using an Agilent HP-5973 Chromatograph (Agilent Technologies, www.agilent.com) equipped with a Shimadzu QP-5000 (Quadruple) mass spectrometer (www.shimadzu.com). The sample was diluted 25 times with acetone, and 1 µL was injected. A fused silica column SPB-1 (30 m/250 mn, film thickness 0.25 µm) coated with polydimethyl siloxane was used. Helium was the carrier gas at a flow rate of 1 mL/min. The injector port temperature was 225 °C, the detector temperature was 250 °C, and the oven temperature was maintained at 60 °C for 1 min and then increased to 225 °C at the rate of 2 °C/min, at which temperature the column was maintained for 5 min. The split ratio was 1:25 and the ionization voltage was 70 eV. Unknown essential oil was identified by comparing its gas chromatography retention time to that of known compounds and by comparison of its mass spectra, either with known compounds or published spectra.

### Insect rearing

The insect colonies were reared under 27 ± 1 °C, 65 ± 5% RH, and darkness condition. *Ephestia kuehniella* flour and mung bean grains were used for rearing of *T. castaneum* and *C. maculatus*, respectively. The second generations of the three insect species were used for experiments.

### Fumigant toxicity

The fumigant toxicity of essential oil on *T. castaneum* and *C. maculatus* were tested in 70 mL glass vials, and 600 mL vials for *E. kuehniella.* In each of them 10 adults (both sexes, male or female, 1–7 days old) were released. No. 1 Whatman filter paper disks (www.whatman.com) were cut to 2 cm in diameter and attached to the undersurface of glass vial screw caps. Filter papers were impregnated with series of pure concentrations of essential oil; for *C. maculatus*: 28.56, 49.98, 92.82, 114.24, and 135.66; for *T. castaneum*: 375, 500, 625, 700, 750, and 1000; for *E. kuehniella*: 0.83, 1.49, 1.99, 2.49, and 3.81). After 24 hours, treated insects were transferred to untreated vials. Four replicates were run for each concentration and control. After 3, 6, 9, 12, and 24 hours from the beginning of exposure, numbers of dead and alive insects were counted, and mortality graphs for all doses were drawn.

### Lethal time (LT_50_) of mortality

For investigation of the lethal time (LT_50_) of *E. cardamomum*, the essential oil on three pests was used from high concentrations obtained from bioassay tests (higher than LC_75_). Selected concentrations (similar bioassay test selection method) were tested, and 4 replications were performed for each dose. The mortality of subjects was counted after 3, 6, 9, 12, 24 and 48 hours, and mortality recording then continued at 3—hour intervals until death of all insects.

**Figure 1.**
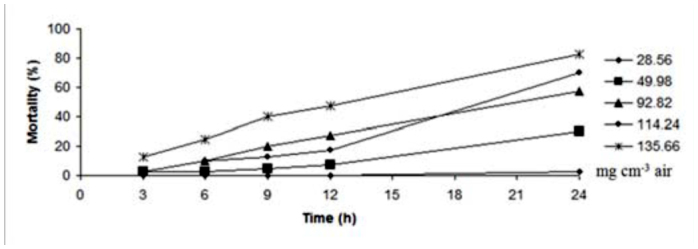
Mortality trend of adults of *Callosobruchus maculatus* exposed to *Elettaria cardamomum* essential oil in different concentrations and times. High quality figures are available online.

**Figure 2.**
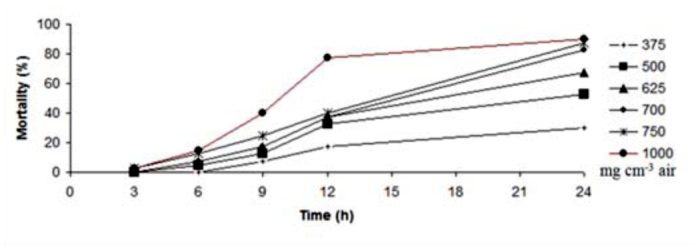
Mortality trend of adults of *Tribolium castaneum* exposed to *Elettaria cardamomum* essential oil in different concentrations and times. High quality figures are available online.

**Figure 3.**
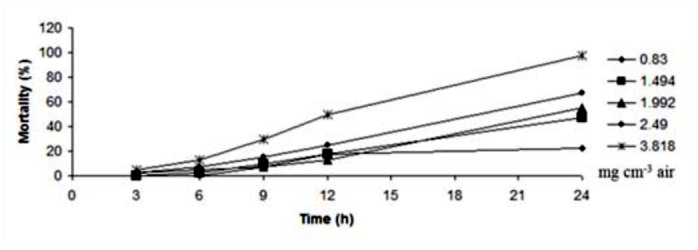
Mortality trend of adults of *Ephestia kuehniella* exposed to *Elettaria cardamomum* essential oil in different concentrations and times. High quality figures are available online.

### Oviposition deterrence of the essential oil on *C. maculatus*

For this study, two pairs (2 males and 2 females) of adults for each concentration and control were selected and put in glass vials. Each vial, containing 5 g of mung bean grains, was treated with 1 mL of acetone solution of different concentrations of essential oil (35.7, 49.98, 57.12, and 64.26 mg/mL). After evaporation of the acetone (∼ 20 min), newly emerged adults of *C.* *maculatus* were transferred to vials. Each concentration was replicated four times. The number of eggs laid in all treatments was recorded after 48 hour.

### Statistical analysis

The lethal concentrations (LC_50_) and lethal time (LT_50_) values of mortality were assessed by Probit analysis using SAS software (SAS Institute 1997). The data from oviposition deterrence were subjected to one—way ANOVA (*p* < 0.05) after checking for normality. Means were compared by Tukey's Studentized Range Test, admitting significant differences at *p* < 0.05. The SAS software was used for all analyses (SAS Institute 1997).

## Results

### Fumigant toxicity

Fumigant toxicity of essential oil of *E. cardamomum* on adults of *C. maculatus*, *T. castaneum*, and *E. kuehniella* after 24 hours is shown in Table 1. There was no mortality in controls.

According to these values, *E. kuehniella* was more sensitive than coleopteran adult insects. Also, *T. castaneum* was more tolerant compared to *C. maculatus.* LC_50_ values of these essential oils after 48 hours are shown in Table 2. After 48 hours LC_50_ values showed that the effect of essential oil was stable and the LC_50_ values after 48 hours were lower than those after 24 hours. Figures 1–3 indicate the mortality trend of these insects after treatment with essential oil of *E. cardamomum.* The highest rate of mortality was seen during 12–24 hours in *C. maculatus* and *E. kuehniella* (Figures 1 and 3), though the highest mortality in *T. castaneum* occurred between 9–12 hours (Figure 2). On the other hand, gradient of mortality in higher doses was more than low doses.

### Lethal time (LT_50_) of mortality

With increasing dose, LT_50_ values of essential oil on *C. maculatus*, *T. castaneum*, and *E. kuehniella* decreased (Table 3). In these tests, *E. kuehniella* sensitivity was higher than two coleopteran insects.

### Oviposition deterrence

The effect of essential oil concentrations of *E. cardamomum* on the females of *C. maculatus* is shown in Table 4. Results showed that the number of eggs laid by females in 48 hours in treatments 57.12 and 64.26 µL/mL was significantly lower than in the control. On the other hand, doses 35.7 and 49.98 µL/mL were not significantly effective (df = 4, 15; *F* = 5.93; *p* < 0.01) (Table 4).

### Chemical constituents of *E. cardamomum*

The results of the chemical analysis are presented in Table 5. Sixteen compounds in the oil were positively identified. In this study 1,8-cineol, α-terpinyl acetate, terpinene, and fenchyl alcohol were found to be the major constituents, accounting for 97% of the total oil.

## Discussion

Plant essential oils have potential as products for control of stored product pests because some of them are selective and have little or no harmful effects on non—target organisms (Isman 1999). Many essential oils are known to possess various bioefficiency effects such as ovicidal, repellent, antifeeding, and biocidal activities against various arthropod pests (Isman 1999; Saxena 1989). For example, neem (*Azadirachta indica*) extract is found to have a variety of biological activities including biocidal activity against nearly 200 arthropod pests without any adverse effects on most non—target organisms (Saxena 1989). Additionally, some plant extracts or phytochemicals are found to be highly effective against insecticide—resistant insect pests (Ahn et al. 1997; Lindquist et al. 1990). The insecticidal constituents of many plant extracts and essential oils against stored product insects are mainly monoterpenoids such as limonene, linalool, terpineol, carvacrol, and myrcene (Ahn et al. 1998; Coats et al. 1991; Regnault-Roger and Hamraoui 1995).

The chemical composition of cardamom varies considerably with variety, region, and age of the product. The content of volatile oil in the seeds is strongly dependent on storage conditions, but may be as high as 8%. In the current study, two compounds (1,8-cineol and α-terpineol) had the highest concentration in the essential oil of *E. cardamomum.* Similar to our results, Olivero-Verbel et al. (2010) stated that the main components found in the volatile oil from *E. cardamomum* were 1,8-cineol (29.7%) and α-terpineol acetate (26.1%). Korikontimath et al. (1999) also showed that the two main components were 1,8-cineol (36.3%) and α-terpineol acetate (31.3%), and reported that the volatile oil of *E. cardamomum* contained about 1.5% α-pinene, 0.2% β-pinene, 2.8% sabinene, 1.6% myrcene, 0.2% α-phellandrene, 11.6% limonene, 36.3% 1,8-cineole, 0.7% γ-terpinene, 0.5% terpinolene, 3% linalool, 2.5% linalyl acetate, 0.9% terpinen 4-01, 2.6% α-terpineol, 31.3% α-terpinyl acetate, 0.3% citronellol, 0.5% nerd, 0.5% geraniol, 0.2% methyl eugenol, and 2.7% trans-nerolidol. In addition, the basic cardamom aroma is produced by a combination of the major components, 1,8-cineole and α-terpinyl acetate (Lawrence 1979).

Additionally, some plant—derived materials are highly effective against insecticide—resistant insect pests (Arnason et al. 1989). In our study, *E. cardamomum* seed essential oil showed good adulticidal activity against *C. maculatus*, *T. castaneum*, and *E. kuehniella*, confirming its usefulness as a candidate for the control of insect pests in stored products. Comparing LC_50_ values of this essential oil after 24 and 48 hours showed that this effect was not reversible and LC_50_ values decreased through time (even after 24 hours). Results showed that moths of *E. kuehniella* were more sensitive than coleopteran adult insects. Also, *T. castaneum* was more tolerant compared to *C.* *maculatus.* Mahmoudvand et al. (2011b) stated that essential oil of *E. cardamomum* had fumigant toxicity on *S. granarious.* Their value of LC_50_ after 24 hours was 220.76 mL L^-1^ air. Comparing our results and those of Mahmoudvand et al. (2011b) suggests that the susceptibility of *C. maculatus* and *E. kuehniella* moths is higher than *S. granarious.* Huang et al. (2000) found that cardamom oil to be a more effective contact poison and fumigant against the adults of *S. zeamais* than those of *T. castaneum.* These findings are similar to those of nutmeg oil (Huang et al. 1997). However, the fumigant toxicity of cardamom oil was six and eight times more potent than nutmeg oil against the adults of *T. castaneum* and *S. zeamais*, respectively (Huang et al. 1997).

Comparison of our results with earlier investigations (Huang et al. 2000) demonstrates different responses of the egg stage and active stages of stored product insects to the *E. cardamomum* essential oil. Our study also shows that cardamom oil successfully prevented the oviposition of *C. maculatus* on mung seeds. Huang et al. (2000) found that cardamom oil dramatically suppressed egg hatching and larval survival of *T. castaneum*, thus showing its ovicidal properties. In addition, it also prevented eggs treated with the oil from developing to the adult stage. Hence, the ovicidal effect of cardamom oil was probably the major factor in the suppression of the development of adults from treated eggs.

For the practical use of the cardamom seed essential oil and test compounds as novel fumigants to proceed, further research is required on the safety issues of these materials for human health. Other areas requiring attention are its mode of action and development of formulations to improve potency and stability, as well as to reduce cost.

In conclusion, results of this study indicate that *E. cardamomum* seed essential oil—derived materials might be useful for managing populations of *C. maculatus*, *T. castaneum*, and *E. kuehniella* in enclosed spaces such as storage bins, glasshouses, or buildings because of their fumigant action, provided that a carrier giving a slow release of active material can be selected or developed.

**Table 1.**
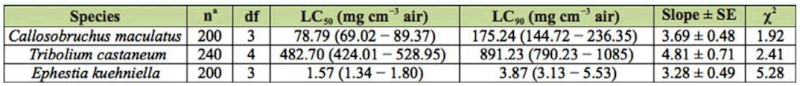
Fumigant toxicity of essential oil of *Elettaria cardamomum* against three stored products pests after 24 hours.

**Table 2.**
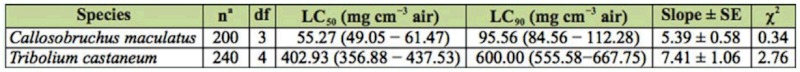
Fumigant toxicity of essential oil of *Elettaria cardamomum* against two stored products pests after 48 hours.

**Table 3.**
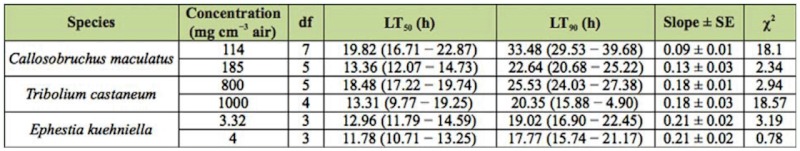
Lethal concentration (LT_50_) of various concentration of essential oil of *Elettaria cardamomum* against three stored products

**Table 4.**
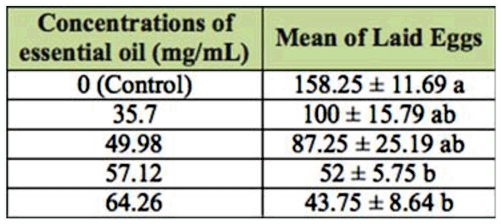
Mean ± SE of oviposition deterrence of *Elettaria cardamomum* essential oil on adult of *Callosobruchus maculatus.*

**Table 5.**
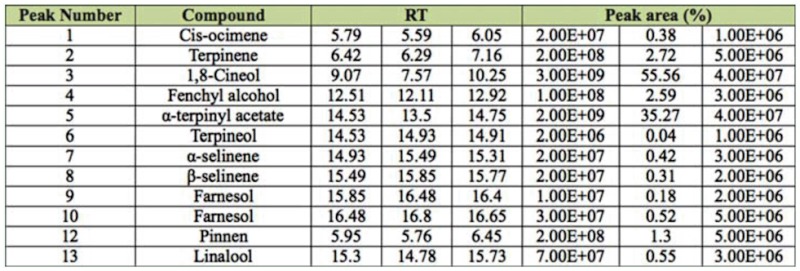
Volatile compounds in steam—distilled oil of the seed from *Elettaria cardamomum* identified by gas chromatography—mass spectrometry. RT: retention time.
